# Clinical diagnostic value of liquid chromatography-tandem mass spectrometry method for primary aldosteronism in patients with hypertension: A systematic review and meta-analysis

**DOI:** 10.3389/fendo.2022.1032070

**Published:** 2022-11-18

**Authors:** Kai-Fang Hua, Yan-Hui Wu, Shi-Ting Zhang

**Affiliations:** ^1^ Department of Endocrinology, Xiang’an Hospital of Xiamen University, Xiamen, China; ^2^ School of Medicine, Xiamen University, Xiamen, China

**Keywords:** meta-analysis, aldosterone, primary aldosteronism, LC-MS/MS, diagnostic test

## Abstract

**Background:**

Primary aldosteronism (PA) is currently considered the most common cause of secondary and endocrine hypertension. Liquid chromatography-tandem mass spectrometry (LC-MS/MS) as a new detection technique has been gradually applied in the diagnosis of PA. However, the diagnostic value of LC-MS/MS methods for PA has not been systematically clinically validated. The aim was to access the diagnostic accuracy, sensitivity, and specificity of LC-MS/MS methods as screening tools in PA.

**Materials and methods:**

A literature search of PubMed, Embase, Medline, Web of Science, Scopus, Science Direct, and Chinese databases was carried out to June 2022 with no language restriction. Data on sensitivity and specificity and other evaluation indicators were extracted and pooled with STATA and Meta-disc software. Heterogeneity was evaluated and meta-regression and subgroup analysis was performed to elucidate sources of heterogeneity.

**Results:**

12 studies of the diagnostic test were suitable and included in the meta-analysis. Pooled sensitivity, specificity, and diagnostic odds ratio were 0.89 (95% CI: 0.83-0.93), 0.87 (95% CI: 0.82-0.91), and 55 (95% CI: 28-110), respectively. Subgroup analysis assessed the diagnostic power of LC-MS/MS based on the type of detection index. ARR and PAC based on LC-MS/MS methods have the higher diagnostic value compared with other indices, diagnostic odds ratios were 121.65 (95% CI: 36.28-407.98) and 49.85 (95% CI: 24.87-99.93). There was considerable heterogeneity among studies.

**Conclusion:**

LC-MS/MS methods had higher accuracy and reliability in the diagnosis of primary aldosteronism. LC-MS/MS-based ARR and PAC can be further promoted and applied in the diagnosis of primary aldosteronism.

## Introduction

Primary aldosteronism (PA) was first described by Jerome Conn in 1955 (1), which is one of the most common causes of secondary hypertension. Compared with essential hypertension(EH), the heart, brain, kidney, and other target organ complications of primary aldosteronism patients are more serious (2), and the incidence of stroke, heart failure, myocardial infarction, and other cardiovascular events is more serious and at higher risk (3). However, PA patients can be “cured” of hypertension by surgery after a clear diagnosis, or the blood pressure can be well controlled by targeted drug therapy (4). Therefore, screening and diagnosis of primary aldosteronism in the hypertensive population are of great significance (5).

The diagnostic process of primary aldosteronism is divided into three steps: screening test, confirmatory test, and type diagnosis (6). Screening is considered indicative of PA on the basis of the plasma aldosterone concentration (PAC) and the aldosterone to renin ratio (ARR) (7). PAC is the absolute value of plasma aldosterone, which has a circadian variation and is affected by location, diet, and renin levels (8). ARR includes the ratio of aldosterone to plasma renin activity or the ratio of aldosterone to plasma renin concentration, which was recommended as the most reliable means of screening for primary aldosteronism by The Endocrine Society Clinical Practice Guideline (9). At present, the screening of primary aldosteronism in clinical practice is mostly based on immunological methods, including radioimmunoassay (RIA) or chemiluminescent immunoassay (CLIA). Radioimmunoassay (RIA) measures renin activity, that is, the level of angiotensinogen converted to angiotensin I per unit of time, which indirectly reflects the level of renin activity in plasma. The renin concentration is measured by chemiluminescence. However, the immunoassay has a non-specific cross-interference of immune response that affects accurate quantification, and the detection values between different laboratories are quite different. The guideline-recommended primary aldosteronism screening for ARR is still based on radioimmunoassay and chemiluminescence immunoassay. Although simple, fast, and low-cost, it also has the problem of radioactive contamination (10).

With the development of detection technology, liquid chromatography-tandem mass spectrometry (LC-MS/MS) is moving toward clinical laboratories as a new gold standard for hormone detection (11). Mass spectrometry(MS) technology detects the mass-to-charge ratio of the substance itself and simultaneously detects the parent ion and product ion, which has high specificity and sensitivity (12). Considered the “gold standard” for hormone detection, mass spectrometry has the advantage of preventing non-specific reactions while avoiding interference from cross-reactions, resulting in higher sensitivity and specificity (13).

The more reliable and specific liquid chromatography-mass spectrometric (LC-MS/MS) method for plasma aldosterone has already replaced plasma aldosterone radioimmunoassay (RIA) in some specialized laboratory centers (14). But the current problem is that the diagnostic value of LC-MS/MS methods has not reached a consensus in the diagnosis of primary aldosteronism (15). This situation may result from the small sample sizes, lack of internationally unified standards, and/or the presence of clinical heterogeneity.

Regarding the limitations of the prior individual studies, to resolve the inconsistencies, and evaluate LC-MS/MS methods for their diagnostic accuracy, there was a need for a systematic approach to analysis. Therefore, we conducted a meta-analysis on the sensitivity and specificity of LC-MS/MS methods according to ARR, PAC, or other detection indices for the diagnosis of primary aldosteronism to assess its Clinical diagnostic value.

## Methods

This systematic review and meta-analysis were conducted by The Cochrane Handbook for Systematic Reviews of Diagnostic Test Accuracy (Version 2.0, 2022).

### PICOS statement

P-patient: adult patients with hypertension for suspected PA. I-index test: LC-MS/MS-based measurement of PAC, ARR, and others. C-comparison: the diagnosis of PA. O-outcome: Primary aldosteronism.

### Search strategy

We systematically searched PubMed, Embase, Medline (*via* Ovid), Web of Science, Scopus, ScienceDirect, and some Chinese databases (CBM, Wanfang Data, and CNKI) without any language limitations, to identify articles published until June 2022 in which LC-MS/MS was performed for a diagnostic test in hypertension patients suspected PA.

We referenced previous similar systematic reviews and searched relevant references list of included primary studies.

To increase the number of results as much as possible and reduce omissions, we developed no P (Population) but I(index) and O (outcome) terms in the search protocol (unpublished). The search formulas were as follows: (“liquid chromatography-tandem mass spectrometry” OR “LC-MS/MS”) AND (“Hyperaldosteronism” OR “primary aldosteronism”). The respective MeSH terms (for PubMed and Medline) or EMTREE terms (for Embase) were also searched in the corresponding database. In the Chinese databases, we also use the above search protocol in Simplified Chinese.

### Study selection and eligibility criteria

We aimed to identify diagnostic studies conducting LC-MS/MS methods of a diagnostic test in hypertension patients for PA. The selection process for inclusion and exclusion of all studies was done independently by two investigators (Hua KF and Wu YH).

This study selection was conducted in four steps, following the Preferred Reporting Items for Systematic Reviews and Meta-Analyses (PRISMA) flow diagram guidelines (16). First, all records retrieved from the seven databases were imported into EndNote version 20 (Thomson Reuters) and duplicates were removed. Second, the titles and abstracts were screened by the two researchers back-to-back to delete irrelevant records. Third, the remaining full-text articles were assessed independently based on the inclusion and exclusion criteria mentioned below. Last, the two authors’ results were compared to check if any articles were misclassified or overlooked. Any discrepancies were recorded and resolved by negotiation. If the researchers failed to reach a consensus, a third investigator (Zhang ST) will step in to discuss and analyze to reach a consensus finally.

The included criteria were as follows:(1) According to the terms of PICOS, studies must mention at least one index detected by LC-MS/MS (PAC, ARR, or others). (2) reference standards must be well-defined for the confirmatory diagnosis of PA among hypertension patients. (3) the studies have essential information to calculate TP, FP, FN, and TN for the 2×2 contingency table. The studies were excluded when they were (1) Case-control studies, Case reports, letters, comments, or reviews (2) based on the methods of chemiluminescent enzyme immunoassay (ELISA) and conventional radioimmunoassay (RIA) (3) without available data or incomplete information. We also excluded all studies that involved healthy volunteers and other situations.

### Data extraction

Eligible studies had the following data extracted independently by two investigators (Hua KF and Wu YH) for the quantitative meta-analysis. We resolved discrepancies through consensus meetings and a third investigator (Zhang ST) to rule on the issues where an agreement could not be reached. Data were extracted from the included studies using a standardized data extraction including study number (No.), the first author’s name, publication year, index detected by LC-MS/MS, the number of PA and EH, cut-off value, sensitivity, and specificity. When multiple diagnostic accuracy values of LC-MS/MS at different cutoffs appeared in the same detection index, we defined the statistically optimal sensitivity and specificity values with the corresponding cutoff value where the Youden index was maximized. The values of a diagnostic 2 × 2 tables (TP, FP, TN, and FN) were also extracted from all primary studies or calculated from available data.

### Methodological quality assessment

The two investigators (Hua KF and Wu YH) used The Quality Assessment of Diagnostic Accuracy Studies 2 (QUADAS-2) to assess the quality of the enrolled articles (17, 18). The QUADAS-2 criteria included nine questions and each question is answered with “high”, “low”, or “unclear”. Different answers represent the degree of risk of bias. We used RevMan 5.4(The Nordic Cochrane Centre, The Cochrane Collaboration, Copenhagen, Denmark) to perform the quality assessment and generate a methodological quality summary graph.

### Statistical analysis

The Statistical analysis process was performed with Meta-Disc 1.4(Metadisc, Madrid, Spain), and Stata SE 15.0(StataCorp LP, College Station, TX, USA). We adopted the bivariate summary receiver operating curve analysis according to the MIDAS module in STATA. Graphs of polled sensitivity and specificity were produced with the MIDAS module. We used the bivariate random-effects regression model proposed by Reitsma (19) for pooling the sensitivity and specificity estimates. The basic principle of the bivariate model is that the sensitivity and specificity of each study are logit-transformed so that they conform to a normal distribution. The bivariate model retains the two-dimensional characteristics of the original data and considers the negative correlation between sensitivity and specificity (20). The comprehensive evaluation value of sensitivity and specificity and the negative correlation value between the two can be obtained by fitting the model (20).

The pooled sensitivity, specificity, positive likelihood ratio (PLR), negative likelihood ratio (NLR), and diagnostic OR (DOR) with 95% confidence interval (CI) were estimated based on the extracted data of true-positive, true-negative, false-negative, and false-positive. Summary receiver-operating characteristics curves (SROC) were drawn using the bivariate model. The closer the curve is to the upper left-hand corner of the SROC curve plot, the better the overall accuracy of the test (21). The area under the curve (AUC) (in this case, area under the SROC curve) represents an overall summary of test performance and displays the trade-off between sensitivity and specificity (22). AUC between 0.90-1.0 is considered as excellent diagnostic accuracy, 0.80-0.90 as good, 0.70-0.80 as fair, 0.60-0.70 as poor, and 0.50-0.60 as fail (21).

Cochran’s Q-statistic and I^2^ are used to assess heterogeneity. Cochran’s Q-statistic test assumes that there is no difference in relative risk within each study. Another indicator of heterogeneity is the I^2^ statistic, which reflects the degree of variation in the study results. I^2 =^ 0% means that the variation is due to random error, and as I^2^ increases, the greater the variability of the results, the more difficult it becomes for random error to explain the variation in the results. Univariable Meta-regression and Subgroup Analysis will be conducted to explore the potential source of heterogeneity if there is significant heterogeneity among studies.

In terms of spearman’s correlation coefficient, logarithm sensitivity and 1-specificity are used to detect threshold effects while a strong positive correlation usually suggests a threshold effect (23). Spearman’s rank correlation coefficient is a technique that can be used to summarize the strength and direction (negative or positive) of a relationship between two variables (24). So, we tested for a threshold effect according to Spearman’s correlation coefficient by the bivariate model.

The Deeks funnel plot asymmetry test was used to evaluate potential publication bias (25). Significant asymmetry is presented when p < 0.10 for the slope coefficient, indicating potential publication bias. If publication bias was present, a sensitivity analysis was performed to explore the sources. P<0.05(two-sided) was considered statistically significant; exact values were P>0.001.

## Result

### Literature search and study characteristics

**Figure 1:**
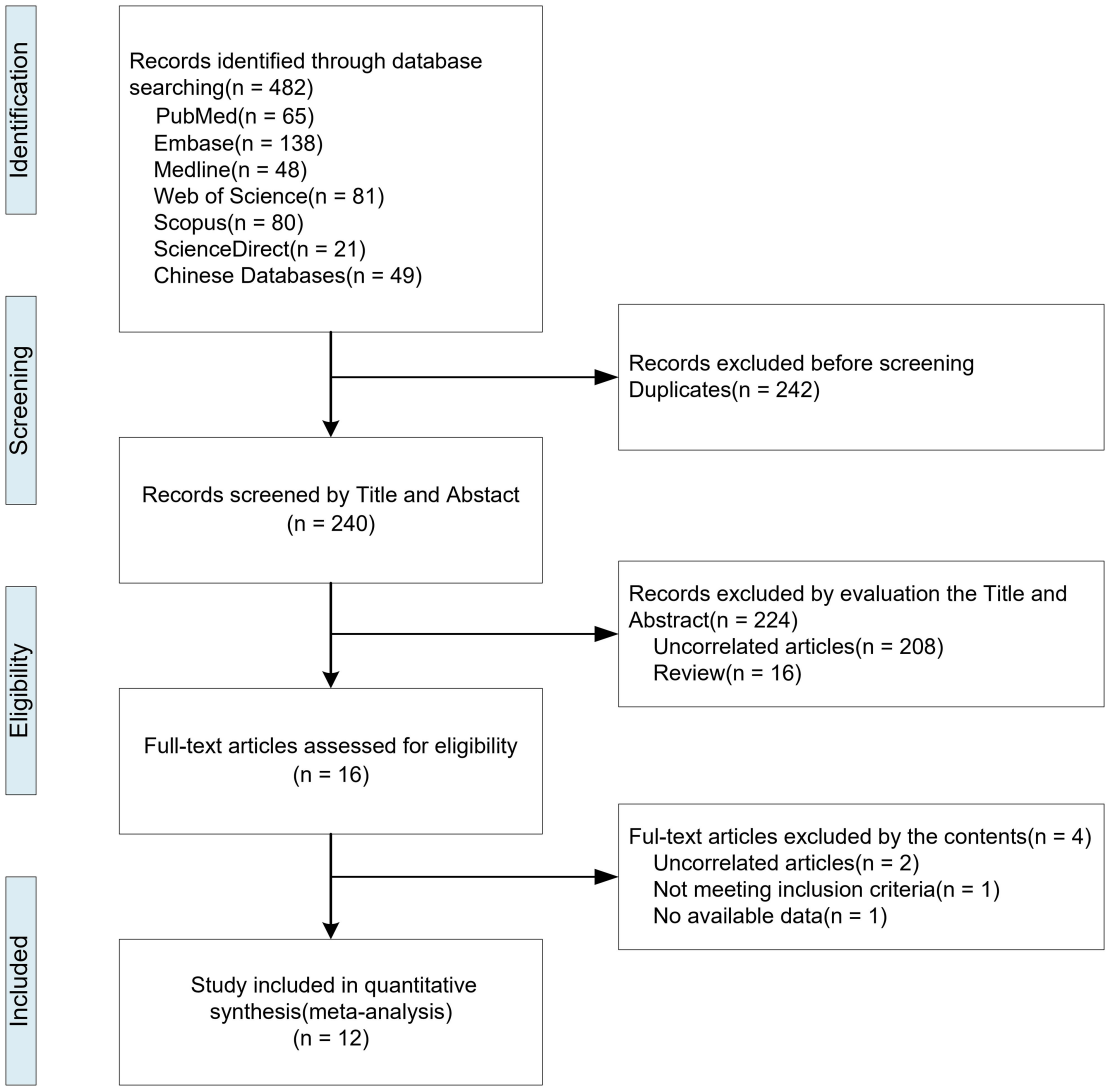
Flowchart of study selection.

The study selection process was illustrated in the flow chart (**Figure 1**). In the first stage of our search strategy, a total of 482 original articles from various databases (**Supplementary Table 1**): PubMed (n = 65), Embase (n = 138), Medline (n = 48), Web of Science (n = 81), Scopus (n= 80), ScienceDirect (n =21), and Chinese Databases (n = 49). In the second stage, after removing 242 duplicates, we screened 240 potentially relevant articles of whom 224 were excluded because of irrelative titles or abstracts. In the third stage, the remaining 16 records were screened in the full text against the eligibility criteria of this review, and four records were excluded. Finally, this systematic review and meta-analysis included 12 records (Supplementary included studies references list), and 20 diagnostic items based on LC-MS/MS methods were extracted for quantitative meta-analysis (**Table 1**).

**Table 1:** Characteristics of the included studies.

No.	Author	Year	Index	PA	EH	Optimal cutoff value	Se	Sp	AUC	TP	FP	FN	TN
1	Baron S	2016	ARR	107	79	45.6 pmol/mUI	1.000	0.939	NA	107	5	0	74
			PAC	107	79	360 pmol/L	0.857	0.951	NA	92	4	15	75
2	Baron S	2018	PAC	82	77	308 pmol/L	0.904	0.915	0.970	74	7	8	70
			ARR	82	77	64.5pmol/mU	0.873	1.000	0.988	72	0	10	77
3	Cheng Z Y	2021	ARR	57	70	6.5 pg/mL	0.942	0.78	0.902	54	15	3	55
			PAC	57	70	24 pg/mL	0.872	0.788	0.876	50	15	7	55
4	Fan Jing	2020	UAC	55	78	11.6 μg/24h	0.509	0.808	0.659	28	15	27	63
			UARR	55	78	4.63(μg/24h)/(μg/L/h)	0.855	0.821	0.906	47	14	8	64
5	Fries C M	2020	PAC	32	67	83 pmol/L	0.969	0.925	NA	31	5	1	62
			ARR	32	67	53 pmol/mU	0.969	0.597	NA	31	27	1	40
6	Fuss C T	2021	PAC	103	84	68ng/L	0.796	0.893	0.908	82	9	21	75
7	Guo Z	2018	ARR	29	8	55 pmol/mU	0.931	0.875	NA	27	1	2	7
8	Juutilainen Auni	2014	ARR	8	31	44 pmol/ng	1.000	0.84	0.970	8	5	0	26
9	Ma W	2019	UARR	60	353	1.11(μg/24h)/(μIU/ml)	0.917	0.890	0.947	55	39	5	314
			ARR	60	353	2.60(ng/dl)/(μIU/ml)	0.933	0.901	0.958	56	35	4	318
			UAC	60	353	7.13 μg/24 h	0.783	0.567	0.725	47	153	13	200
10	Travers S	2019	UAC	64	107	65 nmol/24 h	0.766	0.785	0.864	49	23	15	84
11	Xu Wen	2019	ARR	27	333	NA	0.910	0.963	NA	25	12	2	321
12	Zhao Lin	2019	ARR	143	232	13.84 (ng/dl)/(ng/ml·h)	0.790	0.783	0.837	113	50	30	182
			PAC	143	232	4.29 ng/dl	0.696	0.906	0.807	100	22	43	210

PA, Primary aldosteronism; EH, essential hypertension; Se, sensitivity; Sp, specificity; AUC, area under the receiver operating characteristic curve; TP, true positive; FP, false positive; TN, true negative; FN, false negative.

The characteristic features of the studies included in the meta-analysis were provided in **Table 1**. The 12 included studies comprised 4191 participants (1363 primary aldosteronism patients and 2828 essential hypertension patients), which including 20 different diagnostic tests in total based on different detection indices of LC-MS/MS methods for PA in hypertension patients. There were different study designs in the selected studies. Most were retrospective studies (n = 10), and three were cohort study designs. Different reference standards for primary aldosteronism(as well as the confirmed diagnosis of PA) in the included studies are as follows: Guideline (9) (n = 7), conventional radioimmunoassay(RIA) or chemiluminescent immunoassay(CLIA) (n = 3), surgery or spironolactone medication test (n = 2). The definition of PA was slightly different in each study, but most of them were based on the guideline to discriminate real PA from essential hypertension patients in each article. Only three studies performed further subtype diagnosis in patients diagnosed with primary aldosteronism, of which only two provided diagnostic test data and thus did not allow for quantitative subgroup analysis.

The intra- and inter-assay variations are closely related to the reliability and precision of the diagnostic method. Most of the 12 included studies reported the intra- and inter-assay variation value of aldosterone measurements by LC-MS/MS methods compared with RIA or CLIA. The intra- and inter-assay variation ranged from 2% to 15%. Detailed information on them was provided in **Supplementary Table 2**. The usage of standardized aldosterone samples could also provide an important reference comparison in the diagnostic tests of LC-MS/MS methods. In the included studies, few mentioned the use of standardized aldosterone samples for drawing the standard curve of LC-MS/MS in the main text. But several articles mentioned the stable isotope-labeled internal standard (n = 4) or just healthy people for comparison (n = 2). All 20 diagnostic tests provided sensitivity and specificity, but only 14 studies provided AUC values. Sensitivity ranged from 50.9% to 100% while specificity ranged from 59.7% to 100%.AUC given in the studies ranged from 0.725 to 0.988.

### Quality assessment and publication bias

**Figure 2:**
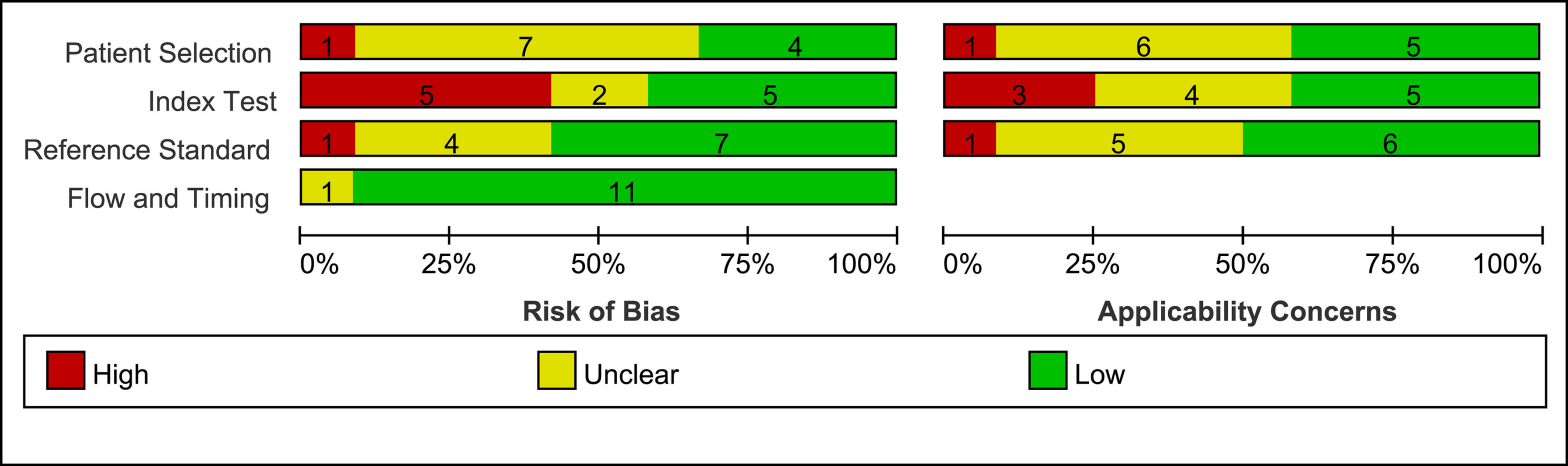
Methodological quality graph for included studies.

The quality assessment of the included 12 studies is summarized in the Methodological quality graph (**Figure 2** and **Supplementary Figure 1**) according to The Quality Assessment of Diagnostic Accuracy Studies 2 (QUADAS-2). The detailed results of the quality assessment were presented in **Supplementary Table 3** by the QUADAS-2 for each item of the 12 reviewed studies. Many of the studies lacked the description of an appropriate interval between index test(s) and reference standard (n = 9). The main high risk of bias was the description of whether to avoid case-control design, and whether the index test results were interpreted without knowledge of the results of the reference standard and vice versa (n = 5). But in general, it showed a good quality of diagnostic studies included in this meta-analysis.

**Figure 3:**
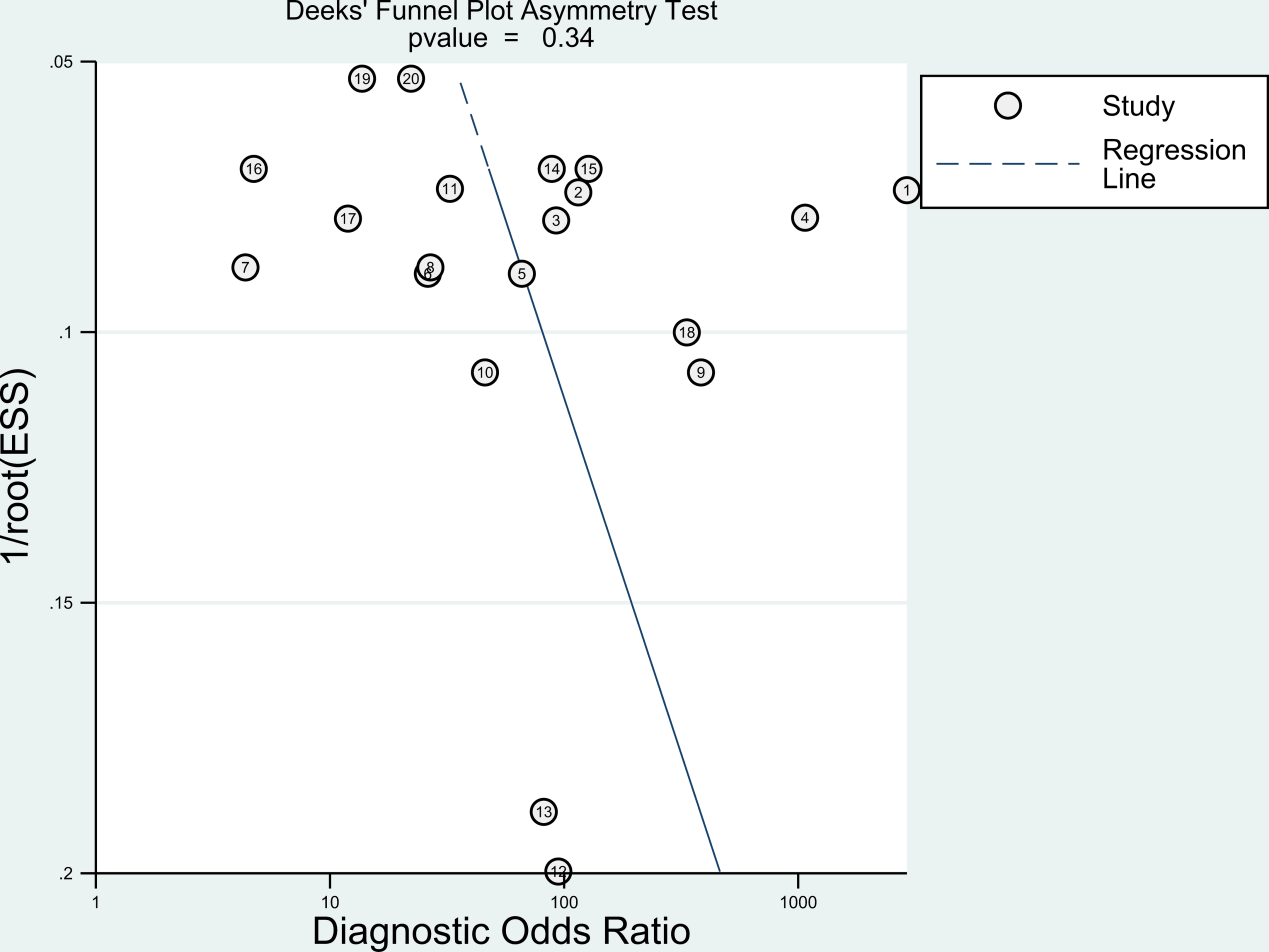
Deeks’ funnel plot asymmetry test.

The Deeks funnel plot asymmetry test of this meta-analysis was not statistically significant (P=0.34), indicating that there is no obvious asymmetry to suggest publication bias (**Figure 3**).

### Diagnostic values of LC-MS/MS methods for PA

**Figure 4:**
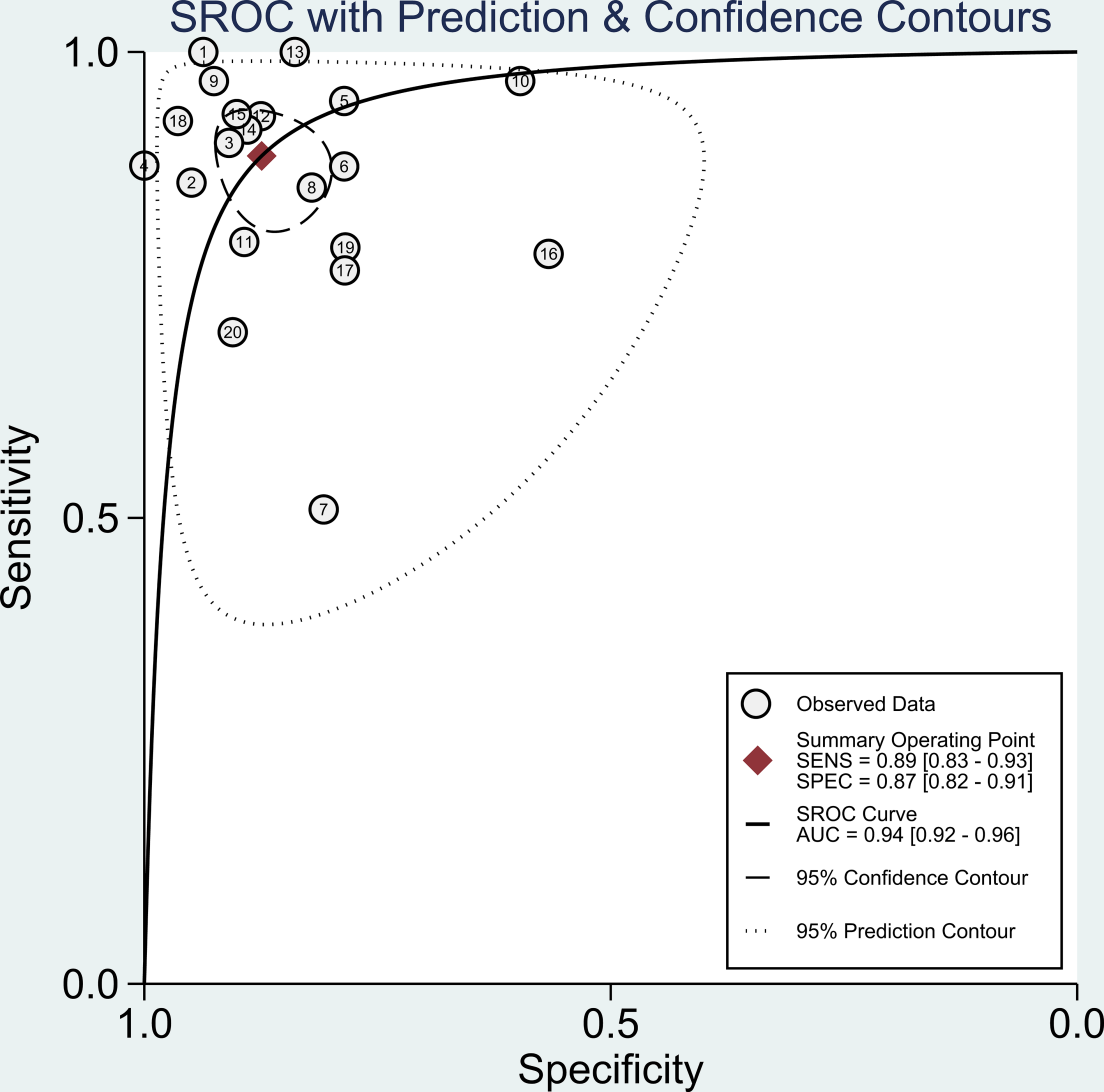
Forest plot of sensitivity and specificity in the diagnosis of LC-MS/MS methods.

**Figure 5:**
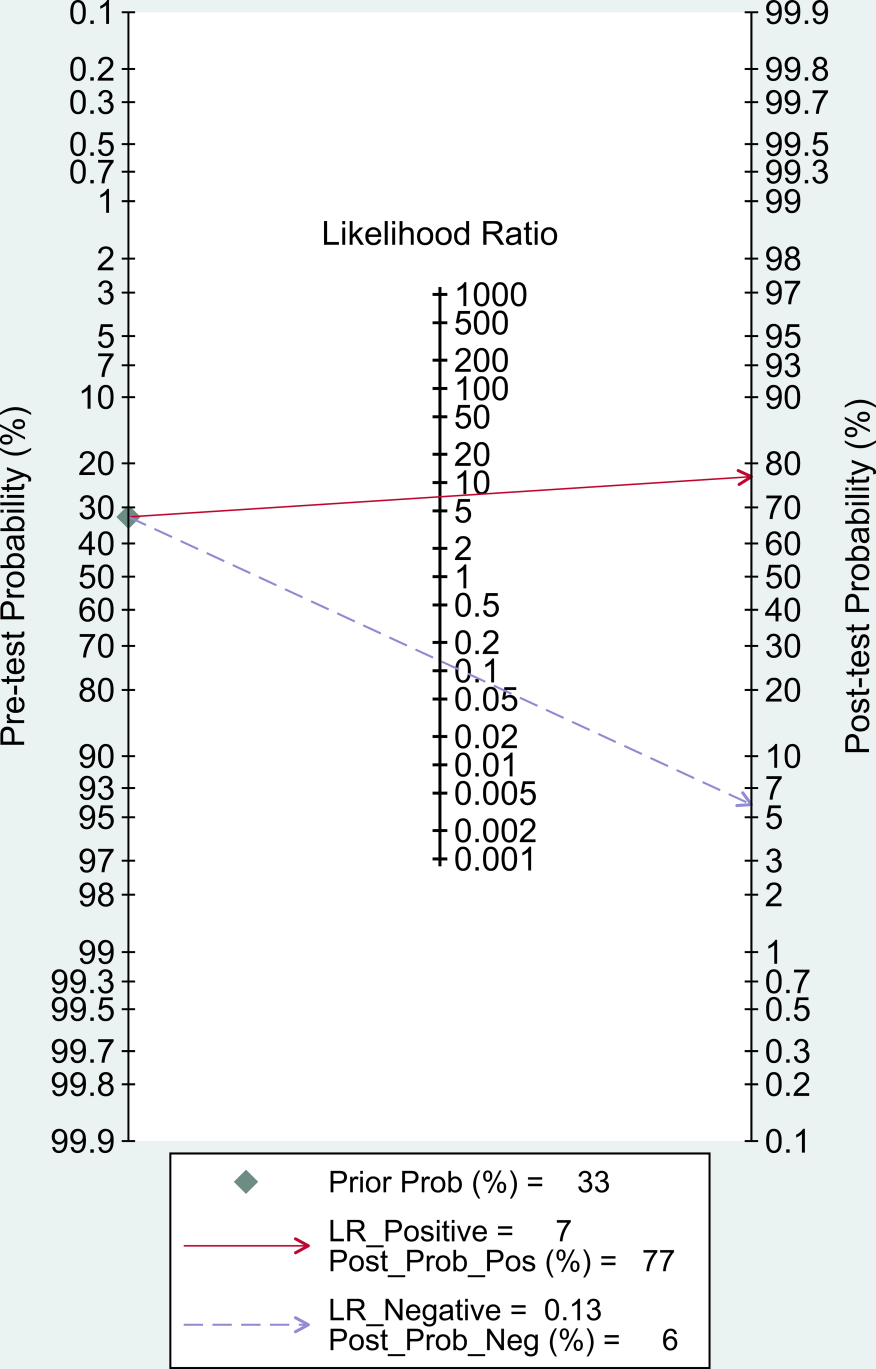
Summary receiver operator characteristic (SROC) curve in the prediction of LC-MS/MS methods for diagnosis of PA.

We pooled the sensitivity and specificity of the included studies to estimate the diagnostic accuracy of LC-MS/MS methods on aldosterone detection in hypertension patients for PA. For this review, the pooled sensitivity was 89% (95% CI: 83%-93%) while the pooled specificity was 87% (95% CI: 82%-91%) (**Figure 4** and **Table 2**). The pooled PLR and NLR were 7.1 (95% CI: 4.9-10.3) and 0.13 (95% CI: 0.08-0.20), respectively (**Table 1**). The pooled diagnostic odds ratio was 55 (95% CI: 28-110) (**Table 2**). The summary receiver operating characteristics (SROC) curve generated by the STATA MIDAS module was shown in **Figure 5**. The area under the receiver operating characteristic curve (AUROC) was 0.94 (95% CI 0.92–0.96) (**Figure 5** and **Table 2**).

**Table 2:** pooled estimate of sensitivity, specificity, PLR, NLR, DOR, DOR and AUROC for LC-MS/MS methods in the diagnosis of PA.

Parameter	Estimate	95% CI
Se	0.89	0.83-0.93
Sp	0.87	0.82-0.91
PLR	7.1	4.9-10.3
NLR	0.13	0.08-0.20
DOR	55	28-110
AUROC	0.94	0.92-0.96

Heterogeneity (Chi‐square): LRT_Q = 57.924, df =2.00, LRT_p =0.000.

Inconsistency (I‐square): LRT_I2 = 97, 95% CI = [94‐ 99].

**Figure 6:**
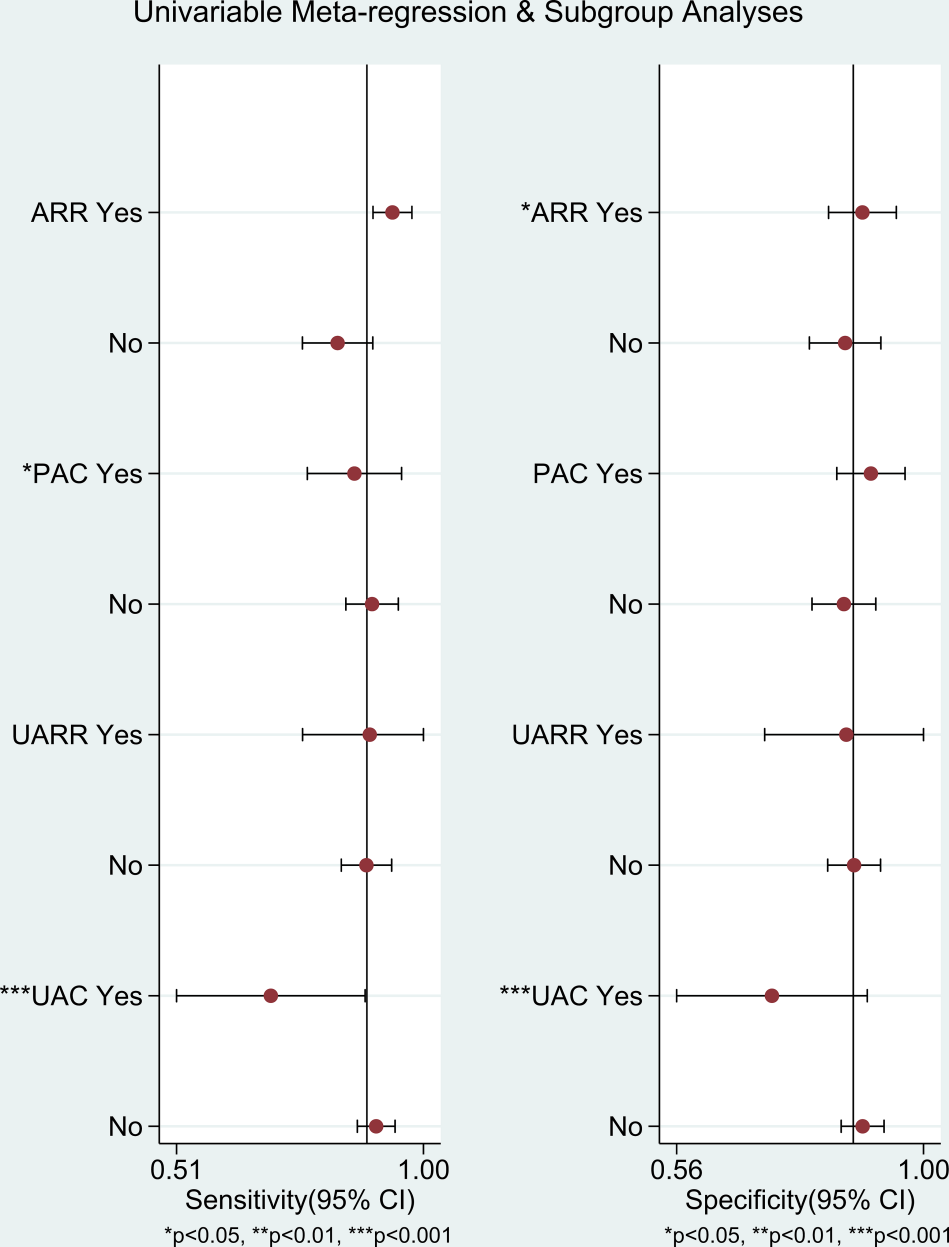
Fagan nomogram of LC-MSMS methods for diagnosis of PA.

In our study, Post-test probability is closely related to the clinical application of diagnostic tests. Given a pre-test probability of 33%, the post-test probability of a positive test result is 77% under this premise. Similarly, a negative likelihood ratio of 0.13 reduces the post-test probability of a negative test result to 6%. (**Figure 6**).

### Test of heterogeneity and threshold effect

The I-squared heterogeneity (tested with Meta-Disc) of sensitivity, specificity, positive likelihood ratio, negative likelihood ratio, and diagnostic odds ratio. is 85.6%, 93.9%, 93.1%, 85.2%, and 84.3%, respectively, (p<0.0001 each). From the information **Table 2** provided, there was significant heterogeneity among the studies. (overall I² for bivariate model 97. 95%, 95% CI 94–99).

Spearman’s correlation coefficient was -0.237 (p = 0.314), indicating no threshold effect (**Table 3**). In addition, there was also no evidence of threshold effect through the plane of the SROC curve plotted the exact estimator of each study (No typical “shoulder-arm” distribution). Therefore, threshold effects were not responsible for the heterogeneity among the studies.

**Table 3:** Analysis of diagnostic threshold.

Var	Coeff.	Std. Error	T	p-value
a	3.881	0.342	11.341	0.000
b (1)	0.122	0.272	0.449	0.6589

Spearman correlation coefficient: -0.237 p-value= 0.314.(Logit (TPR) vs Logit (FPR).

Moses’ model (D = a + bS). Weighted regression (Inverse Variance).

Tau-squared estimate = 1.7895 (Convergence is achieved after 7 iterations).

Restricted Maximum Likelihood estimation (REML).

No. studies = 20.

### Meta-regression and subgroup analysis

**Figure 7:**
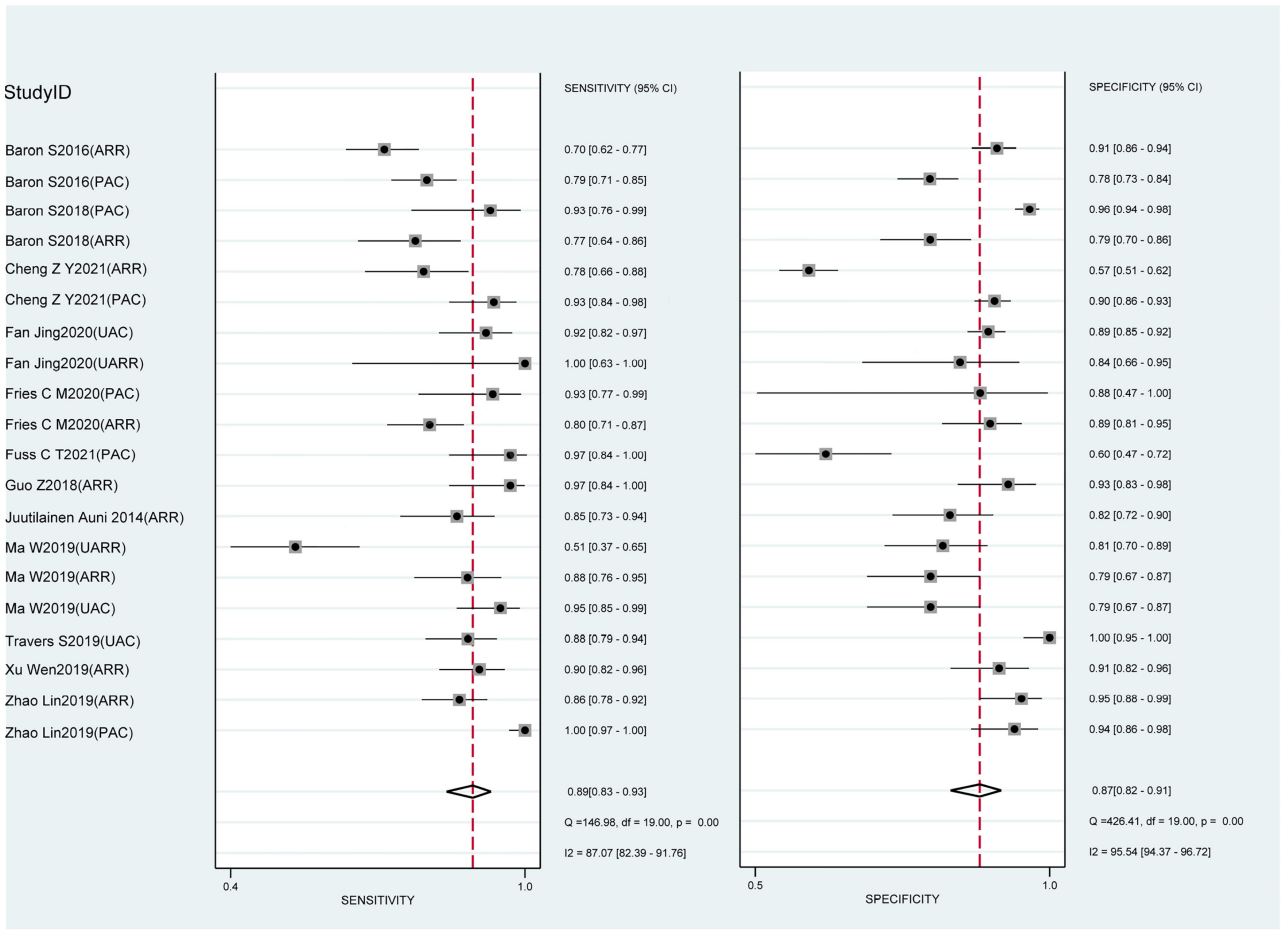
Univariable Meta-regression & Subgroup Analysis.

To identify the source of heterogeneity, we did a meta-regression analysis. Initially, Univariable Meta-regression was conducted for each subgroup and was assessed depending on different detection indices (ARR, PAC, UARR, and UAC). The result (**Figure 7**) implied that there was a certain significant difference in the sensitivity of PAC subgroup and the specificity of ARR subgroup (P<0.05) while strongly significant differences in AUC subgroups (P<0.001).

Further, we conducted subgroup analysis for each index of LC-MS/MS methods by heterogeneity, sensitivity(Se), specificity(Sp), positive likelihood ratio(PLR), negative likelihood ratio(NLR), and diagnostic odds ratio(DOR) reports. The sensitivity and specificity of ARR (aldosterone to renin ratio), PAC (plasma aldosterone concentration), UARR (urinary aldosterone to renin ratio), and UAC (urinary aldosterone concentration) were estimated 0.90 (95% CI: 0.88-0.93) and 0.88 (95% CI: 0.86-0.90); 0.82 (95% CI: 0.78-0.85) and 0.90 (95% CI: 0.87-0.92); 0.89 (95% CI: 0.81-0.94) and 0.88 (95% CI: 0.84-0.91); 0.69 (95% CI: 0.62-0.76) and 0.64 (95% CI: 0.60-0.69), respectively (**Table 4** and **Supplementary Figure 2**–**5**).

**Table 4:** Subgroup analysis for ARR, PAC, UARR and UAC of LC-MS/MS methods.

SubgroupIndex	Number of Studies	Pooled Sensitivity (95%CI)	Pooled Specificity (95%CI)	PooledPLR (95% CI)	PooledNLR (95% CI)	PooledDOR (95% CI)
ARR	9	0.90 (0.88-0.93)	0.88 (0.86-0.90)	7.48 (4.16-13.43)	0.09 (0.04-0.17)	1021.65 (36.28-407.98)
PAC	6	0.82 (0.78-0.85)	0.90 (0.87-0.92)	8.03 (5.38-12.00)	0.17 (0.11-0.28)	49.85 (24.87-99.93)
UARR	2	0.89 (0.81-0.94)	0.88 (0.84-0.91)	6.48 (3.66-11.47)	0.14 (0.07-0.26)	48.49 (15.05-156.20)
UAC	3	0.69 (0.62-0.76)	0.64 (0.60-0.69)	2.52 (1.50-4.22)	0.42 (0.26-0.68)	6.22 (3.34-11.59)

ARR, aldosterone to renin ratio; PAC, plasma aldosterone concentration; UARR, urinary aldosterone to renin ratio; UAC, urinary aldosterone concentration.

With the same order of index detection (ARR, PAC, UARR, and UAC), the positive likelihood ratio(PLR), negative likelihood ratio(NLR), and diagnostic odds ratio(DOR) were calculated 7.48 (95% CI: 4.16-13.43), 0.09 (95% CI: 0.04-0.17), and 121.65 (95% CI: 36.28-407.98) for ARR; 8.03 (95% CI: 5.38-12.00), 0.17 (95% CI: 0.11-0.28), and 49.85 (95% CI: 24.87-99.93) for PAC; 6.48 (95% CI: 3.66-11.47), 0.14 (95% CI: 0.07-0.26), and 48.49 (95% CI: 15.05-156.20) for UARR; 2.52 (95% CI: 1.50-4.22), 00.42 (95% CI: 0.26-0.68), and 6.22 (95% CI: 3.34-11.59) for UAC, respectively (**Table 4** and **Supplementary Figure 2**–**5**).Due to the low number of studies for UARR, the SROC (AUC) in the supplementary figures was not calculated.

## Discussion

### Principal findings

To the best of our knowledge, it was the first study to conduct a systematic review and meta-analysis of the diagnostic value of the LC-MS/MS method for primary aldosteronism in patients with hypertension.

The pooled overall sensitivity, specificity, and DOR had been used to show the overall accuracy of LC-MS/MS in the diagnosis of PA. The pooled sensitivity and specificity of the LC-MS/MS method were 89% (95% CI: 83%-93%) and 87% (95% CI: 82%-91%), respectively, and the AUC of SROC was 0.94 (95% CI 0.92–0.96), showing that LC-MS/MS method had a pretty good diagnostic value. By combining the PLR and NLR, the diagnostic odd ratio (DOR) as a comprehensive diagnostic index is calculated. A DOR value of 55 implied that LC-MS/MS method could be a useful detection technique in PA diagnosis. Further subgroup analysis of each subgroup (ARR, PAC, UARR, UAC) provided evidence that ARR had the highest diagnostic odd ratio (DOR = 121.65) while PAC had the highest Youden index (YI = 0.72) combining the sensitivity and specificity. Therefore, ARR and PAC based on LC-MS/MS had relatively better diagnosis value in clinical practice according to subgroup results. Moreover, in the Fagan nomogram (**Figure 6**) both likelihood ratio and post-test probability also had better differential diagnosis ability. Nevertheless, the calculation of these likelihood ratios is based on dichotomized data, and the diagnosis result of PA is either positive or negative. Considering that plasma aldosterone concentration correlates with the degree of PA progression, calculating likelihood ratios based on multiple cutoffs may provide a more realistic and reliable source of information on the accuracy of the test.

As mentioned earlier, ARR was not only a recommended method by the American guideline, but also proposed by The European Society of Hypertension as the first choice for screening for primary aldosteronism (26). numerous studies have demonstrated ARR has a better sensitivity than the measurement of plasma aldosterone, renin, and potassium concentrations alone (26). However, the lack of accuracy of direct renin concentration (DRC) measurements at low concentrations might affect the ARR and undermine its diagnostic accuracy in most RIA methods. Aldosterone concentrations measured with LC-MS/MS are usually 30% lower than those measured with radioimmunoassay (27), which improved detection precision to some extent.

From the practical clinical perspectives of the Mayo Clinic, given the interpretation of ARR might be confusing due to the wide variation in the lower limits of detection for PRA, it is more practical to use absolute values for PAC and renin (PRA or PRC) (28). However, the promotion and development of this technology are still hindered in clinical practice. Problems such as expensive instruments, high cost of consumables, lack of professional operators, the establishment of laboratory self-built testing methods to be standardized, and the inability to interface with laboratory information systems still need to be further resolved (29).

Considering the heterogeneity in the standards and references for the measurement of PRA or DRC and aldosterone, various thresholds for ARR based on LC-MS/MS are used in different centers. The most recent studies using LC-MS/MS as a reference standard for aldosterone measurements, propose thresholds of 45 pmol/mU (aldosterone in pmol/l and DRC in mUI/l, with a minimum set at 5 mUI/l; the threshold is 1.6 if aldosterone is measured in ng/dl) (26, 30) or a threshold of 55 pmol/mUI without a minimum for DRC (31). Fries et al (27) confirmed consistently lower PAC in patients tested for PA under LC-MS/MS conditions and a screening ARR value of 53 pmol/mUI was found to be most beneficial. In a retrospective study of a Chinese population, random ARR value above 13.84(ng/dl)/(ng/ml·h) can be the cutoff point in suspected PA patients (32).

The reasons for different cutoff values in LC-MS/MS might be attributed to internationally unified standards (33), population sample variation (34), and reference intervals (35).LC-MS/MS analysis involves multi-parameter optimization and equalization, requiring experienced technicians and a perfect quality control system (36). Nowadays, the potential optimal cutoff in LC-MS/MS is not standardized among different laboratories and clinical centers, mainly because of a lack of uniformity in assay methods and in the units used for reporting aldosterone (ng/dL or pmol/L for PAC) and renin (ng/mL/h or pmol/L/min for PRA; ng/L or mU/L for DRC) (37). There is currently no consensus or guideline to guide the clinical application of LC-MS/MS methods, which also limits the unified promotion of LC-MS/MS methods.

The 24-hour urinary aldosterone concentration (UAC) can theoretically reflect the total amount of aldosterone produced by the body in 24 hours. The receptor site and circadian secretion rhythm have less influence which can better reflect the overall secretion of aldosterone than PAC(Yin et al., 2019c). Three studies carried out on UAC have provided evidence that 24-hour urine aldosterone screening for PA also has a pretty good diagnostic accuracy compared to PAC, although a large heterogeneity is suggested in our meta-subgroup analysis (the cutoff values were 7.13μg/24h (38), 11.6μg/24h, and 23.47μg/24h (39), respectively). It is noteworthy that both the absolute value of PAC and the relative value of plasma renin activity (PRA)/plasma renin concentration(PRC) (as well as the ARR) should be reported by the laboratories. PAC combined with ARR as a comprehensive diagnostic index can further improve the diagnostic accuracy in PA, but few studies focused on the combined diagnostic values, especially based on LC-MS/MS methods.

Although it remains unclear whether LC-MS/MS methods can replace immunological methods, the detection of PAC or ARR based on LC-MS/MS is a useful technique that helps to diagnose PA, particularly in hypertension patients.

### Analysis of heterogeneity

Significant heterogeneity had been found in the overall meta-analysis as well as in the subgroup (data not shown). Heterogeneity mainly includes clinical heterogeneity, methodological heterogeneity, and statistical heterogeneity (40). The sources of heterogeneity are manifold. First, Clinical heterogeneity mainly concerns differences in the classification of hypertension patients, quality of medical services, regional ethnicities, and differences in the reference standard for the diagnosis of PA. Second, Methodological heterogeneity maybe arises from different study designs such as cross-sectional studies or cohort studies. Third, Statistical methods are theoretically unlikely to be the main source of heterogeneity in this meta-analysis, given the relatively single statistical evaluation of diagnostic tests. All of the above aspects may be involved in the source of heterogeneity.

### Strengths and weaknesses

Our review and meta-analysis summarized the diagnostic accuracy of LC-MS/MS methods in detecting and diagnosing PA from studies conducted in recent years. This indicated that the results of our review are in sync with the available evidence and that the pooled diagnostic meta-analysis data can provide a reliable and realistic reference for primary aldosteronism on LC-MS/MS methods.

However, there are also a few notable limitations in our review. The studies included in our meta-analysis had substantial heterogeneity as mentioned above, although doing Meta-Regression and Subgroup Analysis between different detection indices, pooling accuracy estimates from two or three studies (such as UAC or UARR) may require further verification. Second, the reliable and precise cutoff values of ARR or PAC based on LC-MS/MS methods are still absent,

Some studies gave the most favorable cut-off value for diagnostic accuracy, and some also give sensitivity and specificity at different thresholds. Cutoff points could not be subsequently validated at the time of meta-analysis pooling, which not only needs to be supported by the bigger populations under broader conditions but also should take into account the healthy population to formulate a reference interval. This is also the key issue that we want to address in our subsequent research work. Last but not least, with the increasing popularity of LC-MS/MS and the concept of precision medicine, guidelines for PA diagnosis and treatment based on the LC-MS/MS analysis are urgently required (36). It is hoped that the widespread use of LC-MS/MS will further facilitate the standardization of aldosterone assay measurements and allow the optimal cutoff values for clinical application as soon as possible.

In conclusion, this systematic review and meta-analysis showed that Liquid chromatography-tandem mass spectrometry (LC-MS/MS) had higher accuracy and reliability in the diagnosis of primary aldosteronism. LC-MS/MS-based ARR and PAC can be further promoted and applied in the detection of primary aldosteronism.

## Data availability statement

The original contributions presented in the study are included in the article/**Supplementary Material**. Further inquiries can be directed to the corresponding author.

## Author contributions

K-FH, methodology, literature selection, data extraction and analysis, and writing. Y-HW, conceptualization, literature searching, data extraction and analysis, and writing. S-TZ, literature checking, data extraction, and analysis writing- reviewing and editing. All authors contributed to the article and approved the submitted version.

## Funding

The work was supported by Special Fund for Introducing High-level Health Talents in Xiamen (code number PM202204140001).

## Conflict of interest

The authors declare that the research was conducted in the absence of any commercial or financial relationships that could be construed as a potential conflict of interest.

## Publisher’s note

All claims expressed in this article are solely those of the authors and do not necessarily represent those of their affiliated organizations, or those of the publisher, the editors and the reviewers. Any product that may be evaluated in this article, or claim that may be made by its manufacturer, is not guaranteed or endorsed by the publisher.
