# Supplementary material for: Clinical diagnostic value of liquid chromatography-tandem mass spectrometry method for primary aldosteronism in patients with hypertension: A systematic review and meta-analysis
Source: Front Endocrinol (Lausanne). 2022 Nov 18;13:1032070. doi: 10.3389/fendo.2022.1032070 (PMC9715607; doi:10.3389/fendo.2022.1032070)
Supplement: Supplementary file 1 [file DataSheet_1.zip › Revised-Supplementary Material Presentation/Supplementary Figure 1-5..docx]

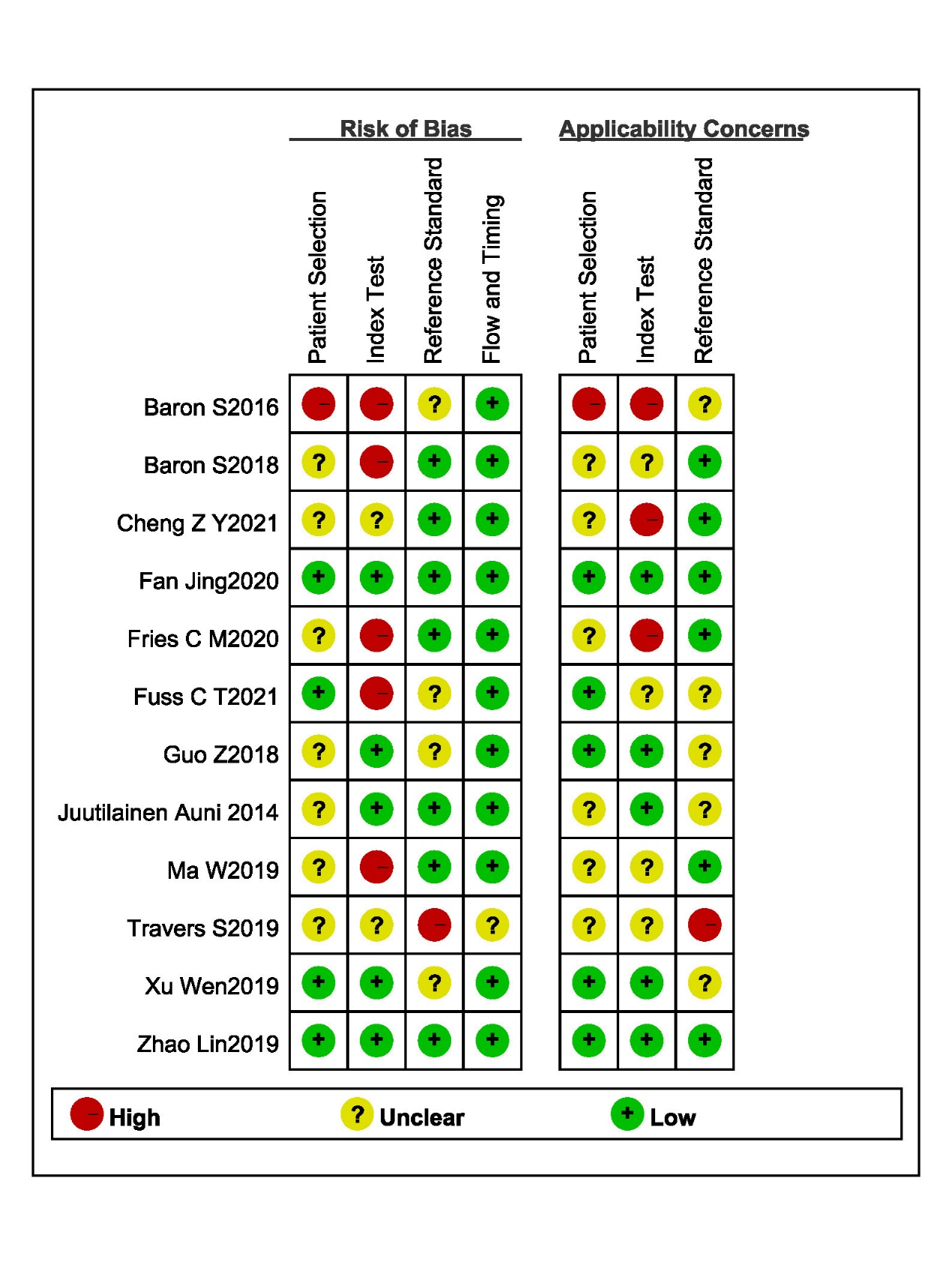


**Supplementary Figure 1**. Methodological quality summary for included studies


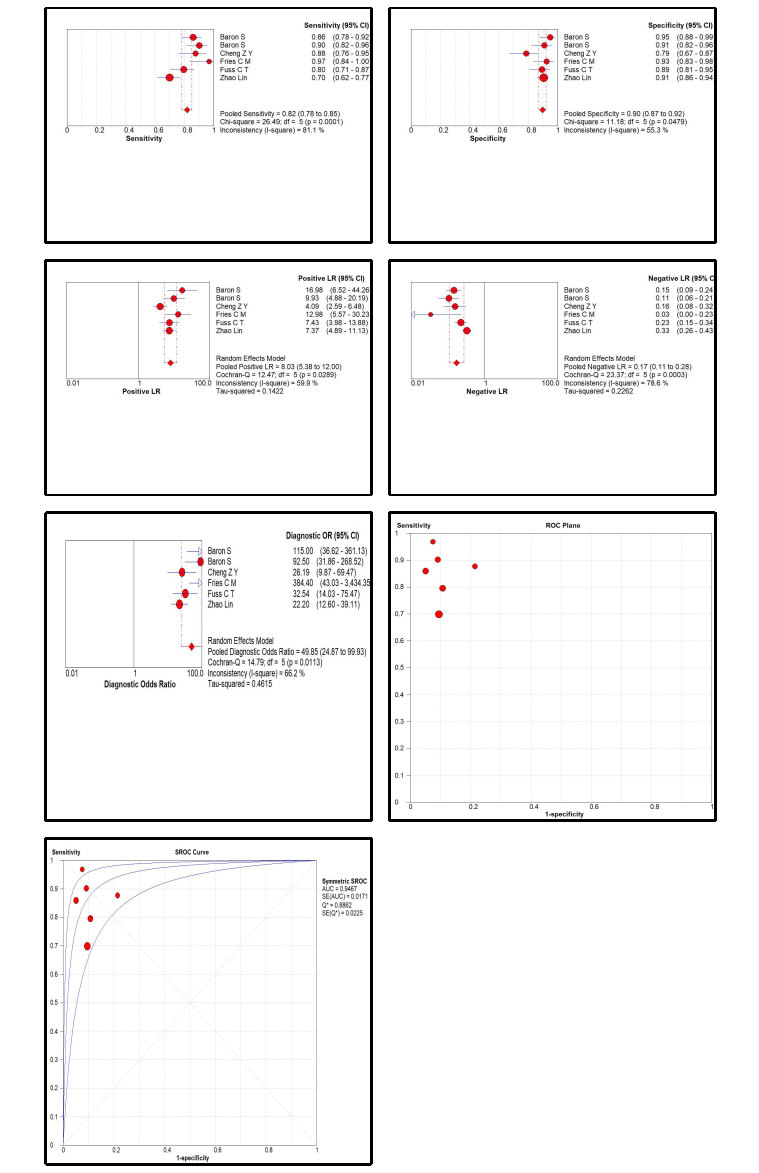


**Supplementary Figure 2.** Forest plots of sensitivity, specificity, PLR, NLR, DOR, SROC curve and ROC Plane for PAC in the diagnosis of PA.


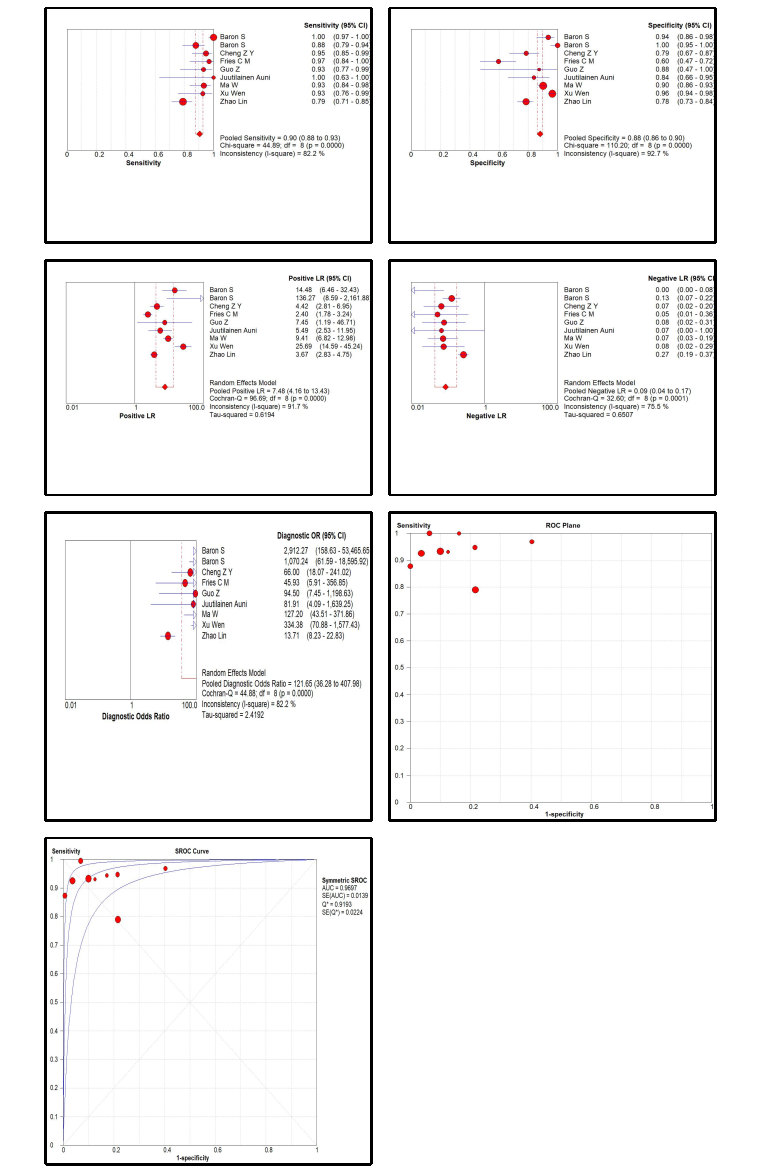


**Supplementary Figure 3.** Forest plots of sensitivity, specificity, PLR, NLR, DOR, SROC curve and ROC Plane for ARR in the diagnosis of PA.


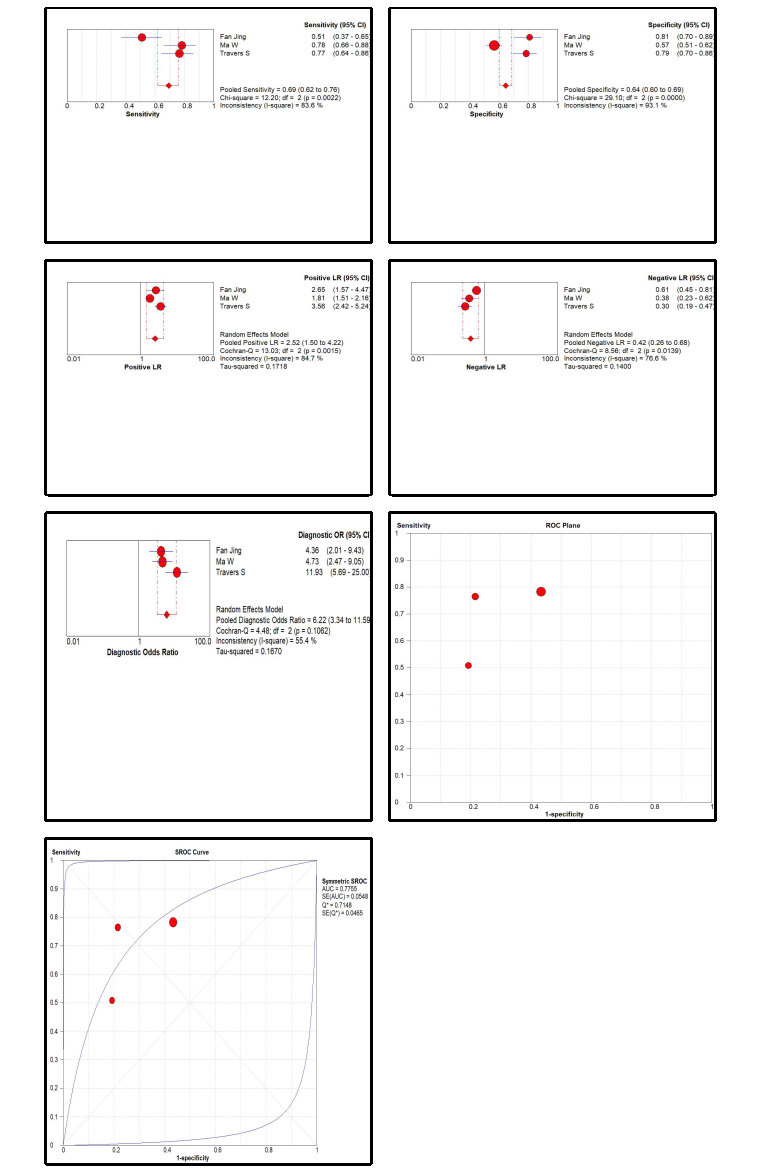


**Supplementary Figure 4.** Forest plots of sensitivity, specificity, PLR, NLR, DOR, SROC curve and ROC Plane for UAC in the diagnosis of PA.


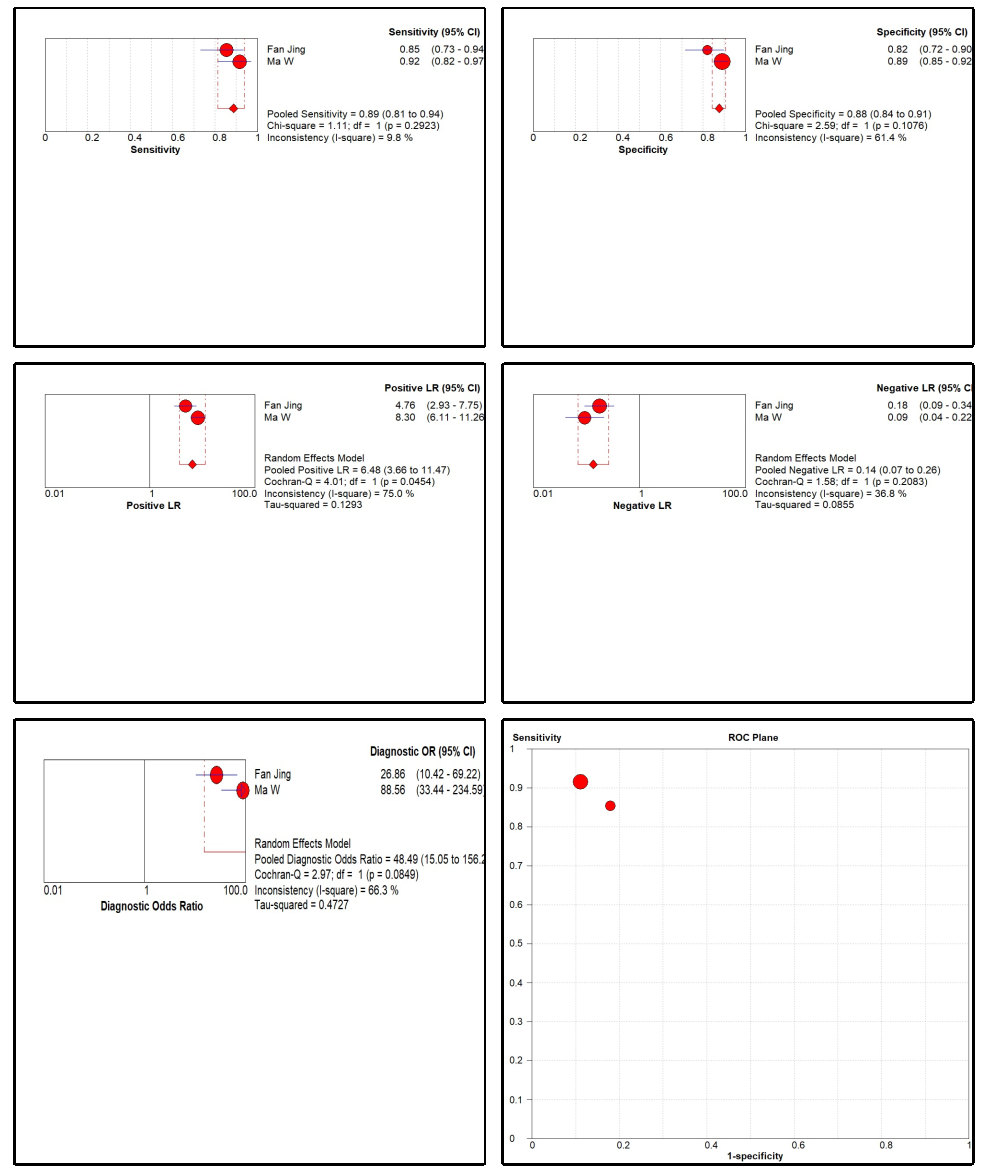


**Supplementary Figure 5.** Forest plots of sensitivity, specificity, PLR, NLR, DOR, SROC curve and ROC Plane for UARR in the diagnosis of PA.
