# Supplementary material for: Clinical diagnostic value of liquid chromatography-tandem mass spectrometry method for primary aldosteronism in patients with hypertension: A systematic review and meta-analysis
Source: Front Endocrinol (Lausanne). 2022 Nov 18;13:1032070. doi: 10.3389/fendo.2022.1032070 (PMC9715607; doi:10.3389/fendo.2022.1032070)
Supplement: Supplementary file 1 [file DataSheet_1.zip › Revised-Supplementary Material Presentation/Supplementary Table 1.Search strategies and search formulas.docx]

**Supplementary Table 1.** Search strategies and search formulas

Database: PubMed, Embase, Medline (via Ovid), Web of Science, Scopus, ScienceDirect and some Chinese databases (CBM, Wanfang Data, and CNKI)

PubMed

| # | Searches | Results |
| --- | --- | --- |
| 1 | liquid chromatography-tandem mass spectrometry [Title/Abstract] | 28680 |
| 2 | LC-MS/MS[Title/Abstract] | 38174 |
| 3 | #1 OR #2 | 54915 |
| 4 | Hyperaldosteronism [MeSH Terms] | 9519 |
| 5 | primary aldosteronism[ Title/Abstract] | 4433 |
| 6 | #4 OR #5 | 10470 |
| 7 | #3 AND #6 | 65 |

Embase

| # | Searches | Results |
| --- | --- | --- |
| 1 | 'liquid chromatography-tandem mass spectrometry':ti,ab,kw | 34017 |
| 2 | 'lc ms':ti,ab,kw | 82478 |
| 3 | #1 OR #2 | 101060 |
| 4 | 'primary hyperaldosteronism'/exp | 7178 |
| 5 | 'primary aldosteronism':ti,ab,kw | 6190 |
| 6 | #4 OR #5 | 8955 |
| 7 | #3 AND #6 | 138 |

Medline

| # | Searches | Results |
| --- | --- | --- |
| 1 | liquid chromatography-tandem mass spectrometry.mp | 28739 |
| 2 | Hyperaldosteronism.mp | 9681 |
| 3 | primary aldosteronism.mp | 4438 |
| 4 | #2 OR #3 | 10509 |
| 5 | #1 AND #4 | 48 |

Web of Science

| # | Searches | Results |
| --- | --- | --- |
| 1 | TS= (liquid chromatography-tandem mass spectrometry) | 32692 |
| 2 | TS=(LC-MS/MS) | 46638 |
| 3 | #1 OR #2 | 65327 |
| 4 | TS=(Hyperaldosteronism) | 3184 |
| 5 | TS= (primary aldosteronism) | 4915 |
| 6 | #4 OR #5 | 6519 |
| 7 | #3 AND #6 | 81 |

Scopus

| # | Searches | Results |
| --- | --- | --- |
| 1 | TITLE-ABS-KEY ("liquid chromatography-tandem mass spectrometry") | 33966 |
| 2 | TITLE-ABS-KEY (“LC-MS/MS") | 48099 |
| 3 | #1 OR #2 | 67252 |
| 4 | TITLE-ABS-KEY ("hyperaldosteronism”) | 13233 |
| 5 | TITLE-ABS-KEY ("primary aldosteronism" ) | 5158 |
| 6 | #4 OR #5 | 13694 |
| 7 | #3 AND #6 | 80 |

ScienceDirect

| # | Searches | Results |
| --- | --- | --- |
| 1 | Title, abstract, keywords: liquid chromatography-tandem mass spectrometry | 17774 |
| 2 | Title, abstract, keywords: LC-MS/MS | 35293 |
| 3 | #1 OR #2 | 43935 |
| 4 | Title, abstract, keywords: hyperaldosteronism | 730 |
| 5 | Title, abstract, keywords: primary aldosteronism | 932 |
| 6 | #4 OR #5 | 1516 |
| 7 | #3 AND #6 | 21 |

Chinese databases (CBM, Wanfang Data, and CNKI)

| Chinese databases | Searches | Results |
| --- | --- | --- |
| CBM | 液相色谱串联质谱法 ＋ 原发性醛固酮增多症 | 6 |
| Wanfang Data | 液相色谱串联质谱法 ＋ 原发性醛固酮增多症 | 12 |
| CNKI | 液相色谱串联质谱法 ＋ 原发性醛固酮增多症 | 31 |
