# Supplementary material for: Clinical diagnostic value of liquid chromatography-tandem mass spectrometry method for primary aldosteronism in patients with hypertension: A systematic review and meta-analysis
Source: Front Endocrinol (Lausanne). 2022 Nov 18;13:1032070. doi: 10.3389/fendo.2022.1032070 (PMC9715607; doi:10.3389/fendo.2022.1032070)
Supplement: Supplementary file 1 [file DataSheet_1.zip › Revised-Supplementary Material Presentation/Supplementary Table 2.The intra- and inter-assay variations.docx]

**Supplementary Table 2.** The intra- and inter-assay variations

| No. | Author | Year | Index | Methods | Intra-assay CV | Inter-assay CV |
| --- | --- | --- | --- | --- | --- | --- |
| 1 | Baron S | 2016 | - | - | - | - |
| 2 | Baron S | 2018 | - | - | - | - |
| 3 | Cheng Z Y | 2021 | PAC | LC-MS/MS | ＜10% | ＜6% |
| 4 | Fan Jing | 2020 | UAC | LC-MS/MS | ≤4.4% | ≤3.2% |
|  |  |  | UAC | CLIA | ≤10% | ≤5% |
| 5 | Fries C M | 2020 | PAC | LC-MS/MS | 3.3–7.4% | 5.2–9.7% |
|  |  |  | PRC | CLIA | 2.5–4.9% | 9.13 ± 2.33% |
| 6 | Fuss C T | 2021 | - | - | - | - |
| 7 | Guo Z | 2018 | PAC | LC-MS/MS | 5.2% | 7.9% |
|  |  |  | PAC | RIA | 5.9% | 7.1% |
|  |  |  | DRC | CLIA | 3.7% | 7.4% |
| 8 | Juutilainen Auni | 2014 | PRA | RIA | 7.5–9.9% | 7.7–11.5% |
|  |  |  | PAC | RIA | 2.3–5.4% | 3.8–15.7% |
| 9 | Ma W | 2019 | UARR | LC-MS/MS | 3.3-4.4% | 2.5-3.2% |
|  |  |  | PAC | CLIA | ＜5% | ＜5% |
|  |  |  | DRC | CLIA | ＜5% | ＜5% |
| 10 | Travers S | 2019 | UAC | LC-MS/MS | 5.6% | 2% |
| 11 | Xu Wen | 2019 | PRA | LC-MS/MS | ＜15% | ＜15% |
| 12 | Zhao Lin | 2019 | PAC | LC-MS/MS | ＜10% | ＜6% |
|  |  |  | PRA | RIA | ＜15% | ＜10% |

PAC=plasma aldosterone concentration. UAC=urinary aldosterone concentration.

PRC= plasma renin concentration. DRC=direct renin concentration

PRA=plasma renin activity. UARR=urinary aldosterone to renin ratio.
