# Supplementary material for: Clinical diagnostic value of liquid chromatography-tandem mass spectrometry method for primary aldosteronism in patients with hypertension: A systematic review and meta-analysis
Source: Front Endocrinol (Lausanne). 2022 Nov 18;13:1032070. doi: 10.3389/fendo.2022.1032070 (PMC9715607; doi:10.3389/fendo.2022.1032070)
Supplement: Supplementary file 1 [file DataSheet_1.zip › Revised-Supplementary Material Presentation/Supplementary Table 3.Results of the QUADAS-2 quality assessment of included studies..docx]

**Supplementary Table 3.**Results of the QUADAS-2 quality assessment of included studies.

| QUADAS2 Items | Patient selection | | | Index test | | Reference standard | | Flow and Timing | | | |
| --- | --- | --- | --- | --- | --- | --- | --- | --- | --- | --- | --- |
|  | Q1 | Q2 | Q3 | Q4 | Q5 | Q6 | Q7 | Q8 | Q9 | Q10 | Q11 |
| Baron S2016 | Y | U | U | N | N | U | Y | U | Y | Y | Y |
| Baron S2018 | Y | U | Y | N | N | Y | Y | U | Y | Y | Y |
| Cheng ZY2021 | Y | U | Y | N | N | Y | Y | U | Y | Y | Y |
| Fan Jing 2020 | Y | Y | Y | Y | N | Y | Y | U | Y | Y | Y |
| Fries C M2020 | Y | N | Y | N | N | Y | Y | U | Y | Y | Y |
| Fuss C T2021 | Y | Y | Y | U | N | U | U | U | Y | Y | Y |
| Guo Z2018 | Y | U | Y | Y | N | U | Y | Y | Y | Y | Y |
| Juutilainen A2014 | Y | U | U | Y | N | U | Y | Y | Y | Y | Y |
| Ma W2019 | Y | U | Y | N | N | Y | Y | Y | Y | Y | Y |
| Travers S2019 | Y | U | U | U | N | U | U | U | Y | Y | U |
| Xu W2019 | Y | Y | Y | Y | N | U | Y | U | Y | Y | Y |
| Zhao L2019 | Y | Y | Y | Y | N | Y | Y | U | Y | Y | Y |

The quality assessment of studies of diagnostic accuracy checklist. Q1: Was a consecutive or random sample of patients enrolled? Q2: Was a case-control design avoided? Q3: Did the study avoid inappropriate exclusions? Q4: Were the index test results interpreted without knowledge of the results of the reference standard? Q5: If a threshold was used, was it pre-specified? Q6: Is the reference standard likely to correctly classify the target condition? Q7: Were the reference standard results interpreted without knowledge of the results of the index test? Q8: Was there an appropriate interval between index test(s) and reference standard? Q9: Did all patients receive a reference standard? Q10: Did patients receive the same reference standard? Q11: Were all patients included in the analysis? Each item can be answered with yes (Y), no (N) or unknown (U).
