# Supplementary material for: Clinical diagnostic value of liquid chromatography-tandem mass spectrometry method for primary aldosteronism in patients with hypertension: A systematic review and meta-analysis
Source: Front Endocrinol (Lausanne). 2022 Nov 18;13:1032070. doi: 10.3389/fendo.2022.1032070 (PMC9715607; doi:10.3389/fendo.2022.1032070)
Supplement: Supplementary file 1 [file DataSheet_1.zip › Revised-Supplementary Material Presentation/Supplementary included studies references list.docx]

**Supplementary included studies references**(Baron et al., 2016, Baron et al., 2018, Ziyun and Zhiqiang, 2021, Jing et al., 2020, Fries et al., 2020, Fuss et al., 2021, Guo et al., 2018, Juutilainen et al., 2014, Ma et al., 2020, Travers et al., 2019, Wen et al., 2019, Lin et al., 2019).

BARON, S., AMAR, L., FAUCON, A., BLANCHARD, A., BAFFALIE, L., FAUCARD, C., PAGNY, J., AZIZI, M. & HOUILLIER, P. 2016. Criteria for primary hyperaldosteronism diagnosis with LC-MS/MS aldosterone measurement. *Journal of Hypertension,* 34**,** e349.

BARON, S., AMAR, L., FAUCON, A. L., BLANCHARD, A., BAFFALIE, L., FAUCARD, C., TRAVERS, S., PAGNY, J. Y., AZIZI, M. & HOUILLIER, P. 2018. Criteria for diagnosing primary aldosteronism on the basis of liquid chromatography-tandem mass spectrometry determinations of plasma aldosterone concentration. *Journal of Hypertension,* 36**,** 1592-1601.

FRIES, C. M., BAE, Y. J., RAYES, N., SANDNER, B., ISERMANN, B., STUMVOLL, M., FAGOTTO, V., REINCKE, M., BIDLINGMAIER, M., MANDY, V., KRATZSCH, J. & FENSKE, W. K. 2020. Prospective evaluation of aldosterone LC-MS/ MS-specific cutoffs for the saline infusion test. *European Journal of Endocrinology,* 183**,** 191-201.

FUSS, C. T., BROHM, K., KURLBAUM, M., HANNEMANN, A., KENDL, S., FASSNACHT, M., DEUTSCHBEIN, T., HAHNER, S. & KROISS, M. 2021. Confirmatory testing of primary aldosteronism with saline infusion test and LC-MS/MS. *European Journal of Endocrinology,* 184**,** 167-178.

GUO, Z., POGLITSCH, M., MCWHINNEY, B. C., UNGERER, J. P. J., AHMED, A. H., GORDON, R. D., WOLLEY, M. & STOWASSER, M. 2018. Aldosterone LC-MS/MS assay-specific threshold values in screening and confirmatory testing for primary aldosteronism. *Journal of Clinical Endocrinology and Metabolism,* 103**,** 3965-3973.

JING, F., XIAOYUN, Z., QILING, F., JUYING, T., SHAOLING, Z., FENG, L., YING, G. & LI, Y. 2020. Urinary Aldosterone Concentration: Its Value in Primary Aldosteronism Screening and Comparison of a LC-MS/MS Assay and a CLIA Assay for Its Determination. *JOURNAL OF SUN YAT-SEN UNIVERSITY(MEDICAL SCIENCES),* 41**,** 563-571.

JUUTILAINEN, A., SAVOLAINEN, K., ROMPPANEN, J., TURPEINEN, U., HäMäLäINEN, E., KEMPPAINEN, J., MOILANEN, L. & PULKKI, K. 2014. Combination of LC–MS/MS aldosterone and automated direct renin in screening for primary aldosteronism. *Clinica Chimica Acta,* 433**,** 209-215.

LIN, Z., JINGJING, J., YING, C., XIAOMU, L., ZHIQIANG, L., WEI, G., XIAOYING, L. & XIN, G. 2019. Clinical significance of LC-MS/MS aldosterone in screening for primary aldosteronism. *Chin J Endocrinol Metab,* 35**,** 927-928-929-930-931-932-933.

MA, W., BIAN, J., LOU, Y., YANG, X., ZHANG, H., ZHOU, X., SONG, L., CAI, J., ZHAO, B., JIANG, E., LIU, W. & CHENG, Y. 2020. Clinical utility value of urinary aldosterone detection by tandem mass spectrometry in primary hyperaldosteronism screening. *Chinese Journal of Laboratory Medicine,* 43**,** 261-266.

TRAVERS, S., BLANCHARD, A., CORNU, E., FAUCARD, C., BAFFALIE, L., AZIZI, M., HOUILLIER, P., AMAR, L. & BARON, S. 2019. Primary aldosteronism: LC-MS/MS assay for 24-hour urine aldosterone determination improves screening. *Journal of Hypertension,* 37**,** e30.

WEN, X., QIAN, Y., FANGJUN, C., JIAJIAN, Q., BAISHEN, P., WEI, G., HUA, B. & BEILI, W. 2019. Method development and clinical application assessment of plasma renin activity (PRA) by liquid chromatography-tandem mass spectrometry (LC-MS/MS). *Fudan Univ J Med Sci*.

ZIYUN, C. & ZHIQIANG, L. 2021. Diagnostic value of liquid chromatography-tandem mass spectrometry in primary aldosteronism. *Chinese Journal of Clinical Medicine,* 28**,** 858-863.
